# PfCSP-ferritin nanoparticle malaria vaccine antigen formulated with aluminum-salt and CpG 1018® adjuvants: Preformulation characterization, antigen-adjuvant interactions, and mouse immunogenicity studies

**DOI:** 10.1080/21645515.2025.2460749

**Published:** 2025-02-04

**Authors:** John M. Hickey, Nitya Sharma, Max Fairlamb, Jennifer Doering, Yetunde Adewunmi, Katherine Prieto, Giulia Costa, Benjamin Wizel, Elena A. Levashina, Nicholas J. Mantis, Jean-Philippe Julien, Sangeeta B. Joshi, David B. Volkin

**Affiliations:** a Department of Pharmaceutical Chemistry, Vaccine Analytics and Formulation Center, University of Kansas, Lawrence, KS, USA; b Division of Infectious Disease, Wadsworth Center, New York State Department of Health, Albany, NY, USA; c Program in Molecular Medicine, The Hospital for Sick Children, Research Institute, Toronto, ON, Canada; d Vector Biology Unit, Max Planck Institute for Infection Biology, Berlin, Germany; e Head of External Research and Development, Dynavax Technologies Corporation, Emeryville, CA, USA; f Departments of Biochemistry and Immunology, University of Toronto, Toronto, ON, Canada

**Keywords:** Malaria vaccine, Alhydrogel, Adju-phos, CpG 1018^**®**^, Adjuvant, PfCSP, Immunogenicity, Formulation, Stability

## Abstract

Circumsporozite protein (CSP), the most abundant surface protein in parasitic *Plasmodium falciparum* (Pf) sporozoite and an attractive target for malaria vaccine design, has been shown to induce protective humoral response in humans. In this work, we characterized and formulated a promising recombinant PfCSP immunogen (155) candidate consisting of two PfCSP epitopes (i.e. junction, NANP repeat) fused to *H. pylori* apoferritin forming a 24-mer nanoparticle. In addition, two N-linked glycans were engineered to mitigate possible anti-apoferritin immune responses, and a universal T-cell epitope was included to further enhance immunogenicity. Physicochemical characterization of the 155 antigen was performed including primary structure, post-translational modifications, conformational stability, and particle size. A competitive ELISA was developed to assess antigen binding to a PfCSP-specific mAb. The *in vitro* antigenicity of the 155 antigen was measured upon formulation with adjuvants, including aluminum-salts (i.e. Alhydrogel^TM^, Adju-Phos^TM^) and the TLR-9 agonist CpG 1018®, when freshly combined and after storage at different temperatures over 3 months. The *in vivo* immunological impact of various adjuvanted formulations of the 155 antigen was investigated in mice. The results support the formulation of 155 with Alhydrogel^TM^ + CpG 1018® adjuvants as a promising recombinant malaria vaccine candidate from both a pharmaceutical and immunological perspective.

## Introduction

Malarial disease in humans is caused by infection from up to five different species of *Plasmodium*, of which *P. falciparum* is the most common. In 2022, an estimated 249 million cases of malaria and 608,000 associated deaths were reported.^1^ Despite substantial annual investments (~4.1 billion USD in 2021) in mitigation and prevention strategies, malaria remains endemic in 85 countries worldwide, particularly in low- and middle-income countries.^1^ In 2021, the World Health Organization (WHO) recommended the use of the malaria vaccine RTS,S/AS01 (Mosquirix^TM^)^2^ with modest and relatively short-lived efficacy (≤36%) in young children.^3^ A second-generation pre-erythrocytic vaccine, termed R21/Matrix-M, was subsequently developed and was recommended for use by the WHO in 2023.^4,5^ However, these two malaria vaccines have less-than-optimal efficacy as well as an inconvenient four-dose vaccination schedule. Development of additional malaria vaccine candidates is ongoing to expand and improve vaccination rates to achieve the WHO’s initiative to reduce malaria transmission by 90% by 2030.^6^

RTS,S/AS01 and R21/MM are recombinant subunit vaccines that stimulate an immune response against the *P. falciparum* circumsporozite protein (PfCSP). PfCSP is the most abundant surface protein in the parasite during the pre-erythrocytic stage and is composed of three domains: an N-terminal heparan sulfate binding domain, a disordered central repeat region that has been shown to induce a protective humoral response in humans, and a T-cell stimulating C-terminal anchor domain.^7–9^ The central region is composed of a singular NPDP motif adjacent to interspersed four-residue repeats (i.e., NVDP, and NANP), referred to as the junctional epitope, which in turn is followed by 35–41 NANP repeats.^9^ The malaria antigen comprising the RTS,S and R21 vaccines consist of 18 NANP repeats from the PfCSP central region and the T-cell C-terminal epitope region fused to Hepatitis B surface antigen (HBsAg), which self-assemble into ~22 nm virus-like particles through the HBsAg scaffold.^10,11^

Numerous novel malaria vaccine candidates are currently under pre-clinical development and clinical testing that target different surface antigens during the three-stage life-cycle of the parasite (i.e., pre-erythrocytic, erythrocytic, sexual) to further improve vaccine efficacy.^12–16^ Recently, a promising malaria vaccine candidate, known as 145S was designed to include both the PfCSP junctional epitope and a short NANP repeat segment that were fused to a nanoparticle scaffold protein (*H. pylori* apoferritin) and a T-cell epitope (PADRE).^17^ Two non-native high mannose glycosylation sites were also genetically engineered into the apoferritin to mitigate a possible anti-apoferritin response and were shown to enhance the PfCSP response. The resulting nanoparticle, when formulated with a novel adjuvant system containing liposomes, QS21, and a toll-like receptor 4 (TLR-4) agonist (3D6AP), was shown to induce a durable protective response in mice.^17^ The observed potent immune responses to 145S was due (in part) to the presence of the junctional region, limited number of NANP repeats, and the omission of the PfCSP C-terminus (unlike RTS,S or R21) to direct the immune response toward the most immunogenic anti-PfCSP epitopes (i.e., junction and NANP repeat).

In this work, we characterized the *in vitro* physicochemical and immunological attributes of a next-generation 145S construct of similar sequence but with improved manufacturability attributes (referred to herein as 155 antigen) in optimized vaccine formulations containing commonly used, low-cost aluminum-salt (alum) adjuvants. Antigen-adjuvant interactions studies were performed between 155 antigen and two commercially available alum adjuvants (Alhydrogel^TM^, AH and Adju-Phos^TM^, AP). These formulations were evaluated both with and without CpG 1018® (CpG) adjuvant, a TRL-9 agonist used in commercial vaccines. The stability profiles of these 155 antigen vaccine formulations were tested both immediately upon preparation (bedside mix) and over 3 months of storage at both 2–8°C and elevated temperatures. The *in vivo* immunogenicity profiles of the 155 antigen formulated with several combinations of these adjuvants were also investigated in mice. The results from these studies are discussed in the context of further optimizing alum+CpG adjuvanted formulations of 155 antigen, both in terms of immunogenicity profiles and pharmaceutical properties, so that this promising malaria vaccine candidate can undergo further pre-clinical testing and eventually clinical trials.

## Materials

The purified 155 antigen in PBS buffer pH 7.0 was aliquoted in 1.5 mL Eppendorf tubes that were stored at −80°C and then thawed on ice prior to use. Both the 155 antigen and the 4493 mAb^7^ (reagent for the competitive ELISA assay) were recombinantly expressed. The aluminum-salt adjuvants (Alhydrogel^TM^, AH and Adju-Phos^TM^, AP) were purchased from InvivoGen (San Diego, CA). The CpG 1018® (CpG) adjuvant was kindly provided by Dynavax Technologies Corporation (Emeryville, CA). All other reagents were purchased from commercial vendors.

## Methods

The expression and purification of the 155 antigen, physicochemical characterization techniques (i.e. SDS-PAGE, Intact Protein Mass Spectrometry, Differential Scanning Calorimetry (DSC), Dynamic Light Scattering (DLS), Zeta Potential, and antigen-antibody binding assay (Competitive ELISA)), sample preparation and stability studies, mouse immunogenicity studies, end point titer analysis, Pf sporozoite hepatocyte traversal assay, and statistical analyses are described in detail in the Supplemental Material.

## Results

### Physicochemical characterization of the recombinant glycoprotein nanoparticle (155 antigen)

**Figure 1.**
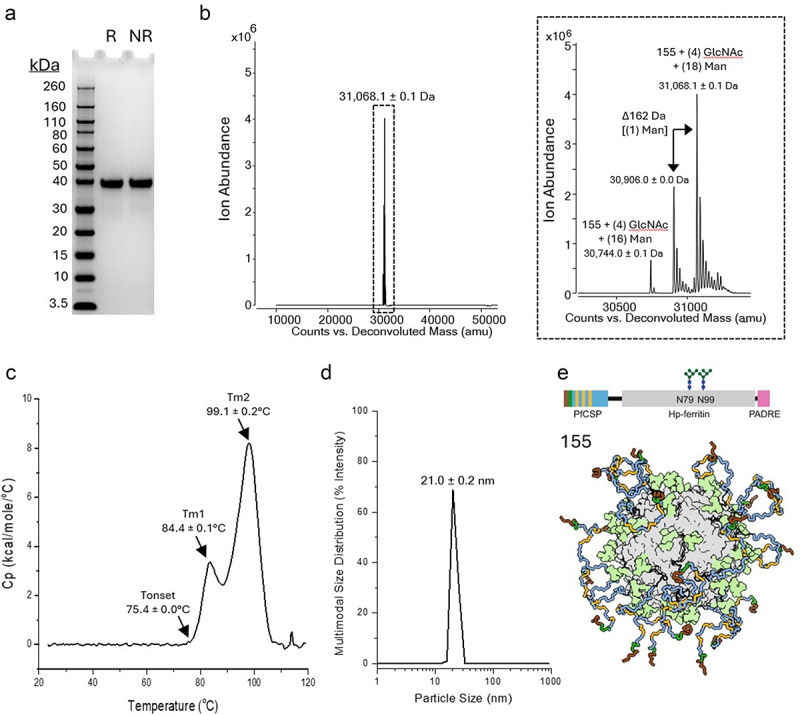
Physicochemical analyses of recombinant glycoprotein nanoparticle antigen 155. (a) Primary structure of the 155 antigen as measured by (A) SDS-PAGE with representative gels under reducing (R) or non-reducing (NR) conditions shown, and (b) Intact mass analysis with representative deconvoluted intact mass spectrum (5–55 kDa) of non-reduced monomer shown with indicated mean molecular weight value ± SD (*n* = 3, three independent samples each measured once). The 29–32 kDa region (boxed figure) was enlarged for easier visualization of the high mannose glycosylated 155 antigen species. (c) Conformational stability of the 155 antigen as measured by DSC with representative thermogram shown. Thermal onset (Tonset) and melting temperatures (Tm1 and Tm2) mean values ± range (*n* = 2, two independent samples each measured once). (d) Particle size analysis as measured by DLS (representative multimodal size distributed and intensity weighted analysis shown) with mean hydrodynamic radius values ± SD (*n* = 3, three independent samples each measured once). (e) Linear representation of a 155 monomer (top) and 3-dimensional cartoon of a 155 antigen nanoparticle (bottom) with the PfCSP junction epitope shown in red, dark green and yellow sticks, the PfCSP NANP repeat shown in blue stick, the engineered N-linked glycans shown in light green surface, and the apoferritin shown in gray surface. The PADRE linear peptide (pink) is encapsulated inside the nanocage and not visible. This image was adapted from Ref.^17^ under the Creative Commons Attribution 4.0 International License.

Physicochemical analysis of the 155 antigen in solution was performed first to provide baseline data on the antigen’s structural properties prior to formulation studies with one or multiple adjuvants. The primary structure of the 155 antigen was initially assessed by SDS-PAGE analysis under reducing or non-reducing conditions (Figure 1a). One prominent band was observed under either treatment condition, which migrated slightly below the 40 kDa MW marker, and was larger than the calculated primary sequence mass of a 155 antigen monomer (~27.3 kDa). The increased mass of the 155 antigen by SDS-PAGE analysis was likely due to the presence of high mannose glycans at two engineered N-linked glycan sites on the apoferritin region of the antigen.^17^ To confirm this hypothesis, 155 antigen samples were subjected to intact mass analysis. Multiple species were observed in the deconvoluted mass spectrum of a non-reduced 155 antigen monomer (Figure 1b). The mass of the most abundant species (31,068.1 ± 0.1 Da) was consistent with the mass of the 155 antigen’s primary sequence and the addition of two glycans consisting a total of 4 N-acetylglucosamine and 18 mannose moieties. Less abundant lower MW species were also observed that were separated by 162 Da (single mannose) and indicated some small variation in the high mannose glycan chain lengths, which is well known to occur in glycoproteins produced in kifunensine-treated CHO cells.^18^

The higher-order structure of the 155 antigen was evaluated in terms of overall conformational stability, particle size and surface charge analyses. For the former, the 155 antigen in HBS formulation buffer (10 mm HEPES, 150 mm NaCl (HBS) pH 7.0) was subjected to differential scanning calorimetry (DSC) analysis with temperature ramping between ~20° and 120°C (Figure 1c). Two endothermic structural transitions were observed at relatively high temperatures in the 155 DSC thermogram, with an initial onset thermal temperature (Tonset) value of 75.4 ± 0.0°C followed by two thermal melting temperatures of 84.4 ± 0.1°C (Tm1) and 99.1 ± 0.2°C (Tm2). The two observed structural transitions in the DSC thermogram of the 155 antigen were possibly due to structural alterations of the nanoparticle (transition at 84.4°C) and the ferritin portion of the 155 antigen monomer (transition at 99.1°C), but these transition assignments require experimental confirmation. Next, the particle size of the 155 antigen nanoparticle in solution and the potential presence of aggregates were measured by dynamic light scattering. The mean hydrodynamic diameter (from multimodal size distribution intensity analysis) and polydispersity index of the 155 antigen nanoparticle were determined to be 21.0 ± 0.2 nm and 0.05 ± 0.03, respectively (Figure 1d). This result is consistent with (1) the particle size range of other ferritin-based vaccine candidate nanoparticles (20–40 nm),^19^ and (2) no notable aggregation being observed. Finally, the computationally predicted isoelectric point (pI) value of the 155 antigen is 5.1, which suggests that the protein should have an overall negative charge in the formulation buffer (HBS pH 7.0). To confirm the charge of the protein when assembled into a nanoparticle, the surface charge was measured by zeta potential analysis. Results showed the 155 antigen nanoparticles in HBS pH 7.0 have a net negatively charged surface with a zeta potential value of −7.9 ± 0.6 mV. Overall, these results demonstrate that the 155 antigen is of high purity, consisting of an apoferritin nanoparticle of expected size displaying 24 copies of the negatively charged PfCSP antigen of expected composition along with relatively homogenous high-mannose oligosaccharides. A schematic of the primary and higher-order structure of the 155 antigen, a recombinant glycoprotein nanoparticle, is displayed in Figure 1e.

### Interactions of 155 nanoparticle antigen and CpG adjuvant with colloidal suspensions of either AH or AP aluminum-salt adjuvants

**Figure 2.**
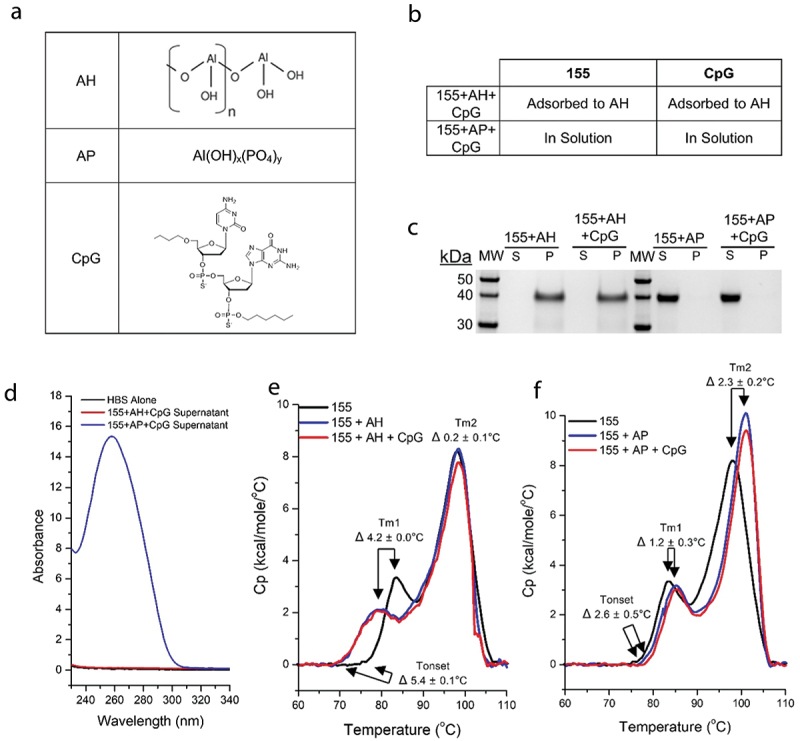
Nature of the interactions between 155 antigen and aluminum-salt adjuvants and CpG.

The physical interactions between the 155 antigen, CpG 1018® (CpG) adjuvant, and the two aluminum-salt adjuvants (Alhydrogel^TM^, AH; Adju-Phos^TM^, AP) were evaluated *in vitro* to elucidate their potential effects on mouse immunogenicity and storage stability profiles. The negative surface charge of 155 antigen nanoparticle in HBS pH 7.0 (zeta potential of~ −8 mV see schematic, Figure 1e), along with the negative charge of the CpG oligonucleotide (see Figure 2a for schematic of adjuvant structures) suggests that both would bind the colloidal suspensions of the positively charged surface of AH but remain in solution in the presence of negatively charged surface of AP, if the prominent antigen-aluminum salt adjuvant interaction forces are electrostatic in nature. In fact, previous studies have shown that CpG adsorbs to AH (zeta potential of ~ +22 mV) but remains in solution with AP (zeta potential of ~ −18 mV), at neutral pH.^20^ After the 155 antigen was incubated with either of the two aluminum-salt adjuvants, and CpG, samples were centrifuged to pellet the aluminum particles and binding interactions were determined. SDS-PAGE of the supernatant and pellet fractions indicated that the 155 antigen fully adsorbed to AH and remained in solution in the presence of AP (Figure 2b,c). As expected, the target concentration of CpG (0.6 mg/mL) was measured in solution in the AP formulation but not observed in AH supernatant fractions using UV-visible spectroscopy. These results demonstrate that CpG had adsorbed to AH but remained in solution in the presence of AP (Figure 2d).

(a) The representative images of adjuvants including composition of the AH (adapted from Ref.^36^ with permission) and AP (adapted from Ref^24^ under the Creative Commons Attribution 4.0 International License) colloidal suspensions, and the structure of the CpG (adapted from Ref.^37^ with permission). (b) Summary table of observed binding interactions of 155 antigen and CpG with two different aluminum-salt adjuvants, AH (Alhydrogel™) or AP (Adju-Phos™). (c) Representative antigen-alum binding data measured by reduced SDS-PAGE analysis of the supernatant (S) or pellet (P) fractions. (d) Representative CpG-alum binding data measured by UV-visible spectra of buffer blank (black trace) or supernatant of 155+AH+CpG (red trace) or 155+AP+CpG (blue trace). (E, F) Conformational stability analysis of the 155 antigen as measured by DSC. (e) Representative thermograms of the 155 antigen in solution (black trace), adsorbed to AH (blue trace) or adsorbed AH to with CpG (red trace) in HBS pH 7.0 buffer. (f) Representative thermograms of the 155 antigen in solution (black trace), in presence of AP (blue trace) or in presence of AP with CpG (red trace) in HBS pH 7.0 buffer. Indicated ΔTonset, ΔTm1, and ΔTm2 mean values ± range (*n* = 2, two independent samples each measured once).

The overall conformational stability of the 155 antigen in the presence of CpG and aluminum-salt adjuvants was investigated using DSC. Compared to unadjuvanted 155, the Tonset and Tm1 values of the 155 antigen adsorbed to AH were ~5°C and ~4°C lower, respectively (i.e., ΔTonset and ΔTm values as shown Figure 2e). This result indicated that the adsorption of the 155 antigen to the surface of AH had a destabilizing effect upon exposure to high temperatures. In the presence of AP, however, Tonset, Tm1, and Tm2 values of 155 increased by 1–3°C compared to unadjuvanted 155 antigen (i.e., ΔTonset and ΔTm values as shown Figure 2f), a result demonstrating no destabilizing effect of AP at high temperatures. The presence of CpG did not influence the stability profile of the 155 antigen either adsorbed to AH or in solution with AP during DSC analysis. In summary, these results indicated that both the 155 antigen and CpG are fully adsorbed to AH, while both remained in solution with AP. The thermal stability of the 155 antigen was not affected by the addition of CpG (in the presence of either AH or AP), and minor destabilizing effects were observed by the presence of the two aluminum-salt adjuvants only when the antigen is bound to adjuvant and at relatively extreme temperatures (>65°C).

### Competitive ELISA development to measure in vitro antigenicity of 155 formulated with adjuvants

A competitive ELISA was developed to quantify the binding of a PfCSP-specific mAb (4493)^21^ to the 155 antigen, both as a purified solution (bulk) and when formulated in the presence of aluminum-salt adjuvants (AH, AP) and CpG (drug product). Due to the colloidal nature of AH and AP, a competitive ELISA format allows for monitoring of antigen-antibody binding with adjuvanted 155 antigen without the need for dissolving the alum or desorbing the antigen from the alum surface.^20^ The competitive ELISA was evaluated for analytical suitability parameters including linearity, accuracy, precision, and stability indication as described below.

**Figure 3.**
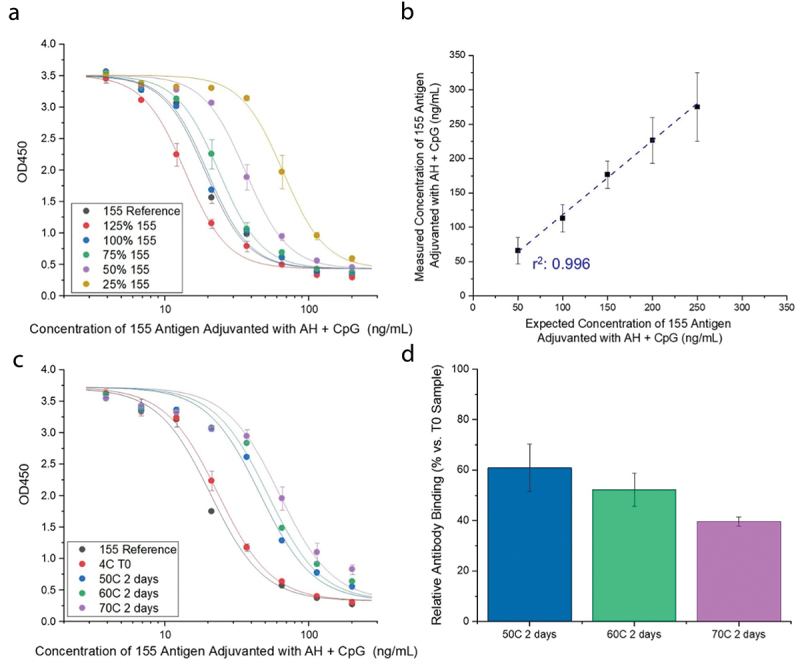
Development of competitive ELISA to measure ability of antigen-specific mAb to bind 155 antigen formulated with aluminum-salt and CpG adjuvants. (a) Competitive ELISA antibody-antigen binding curves and (b) corresponding relative percent antibody binding values for samples containing different concentrations of 155 antigen to demonstrate assay linearity. Error bars denote SD from *n* = 6 (six independent samples each measured once). The measured concentrations of 155 antigen were fit using a linear regression analysis (blue dashed line). (c) Competitive ELISA curves antibody-antigen binding for samples of formulated 155 antigen after thermal stress to establish stability-indicating nature of assay. Samples contained 0.2 mg/mL 155 antigen adsorbed to 3.0 mg/mL AH with 0.6 mg/mL CpG in HBS pH 7.0 buffer at time 0 (T0) and after 2 d of storage at 50°, 60°, or 70°C. (d) Corresponding relative percent antibody binding values of the thermal stressed samples relative to the T0 sample. Error bars denote SD for *n* = 4 (two independent samples measured in duplicate).

Linearity and precision of the assay were demonstrated by analyzing 155+AH+CpG samples prepared at 25–125% of the 200 ng/mL target 155 antigen concentration (Figure 3a). The measured average concentration between two analysts was determined with a linear response (r^2^: 0.996) observed displaying a percentage coefficient of variation of less than 20% (Figure 3b). Stability indication was established where the 155 antigen adsorbed to AH with CpG was analyzed immediately at time 0 (T0) or after storage at different temperatures (50°, 60°, or 70°C) for 2 d. These three stress temperatures were chosen based on initial experiments at varying incubation times to identify extreme conditions to destabilize the antigen to facilitate method development (data not shown). After 2 d of incubation, a large right-shift in the sigmoidal dose-response curve was observed with AH-adjuvanted 155 antigen samples stored at ≥50°C (Figure 3c). Relative to a 155 antigen control sample (155 antigen freshly adsorbed to AH with CpG), antibody binding in the T0 (4°C) 155 antigen sample trended slightly lower (<13%) but was within the variability of the assay (~20%). In thermally stressed samples (2 d at 50°, 60°, or 70°C), a ~ 40%, ~50%, or ~60% loss in PfCSP mAb binding was measured, respectively (Figure 3d).

Following the establishment of competitive ELISA to measure the amount of PfCSP-specific antibody binding to the 155 antigen (formulated with different adjuvants), we monitored and compared the *in vitro* antigenicity of multiple 155 antigen formulations during preparation (bedside-mix) and upon storage at different temperatures over 3 months. We also compared the *in vitro* antigenicity results of some of the 155 antigen stability samples with *in vivo* immunogenicity readouts in mice as described in the next sections.

### Immunogenicity profiles of 155 antigen formulated with or without adjuvants

**Figure 4.**
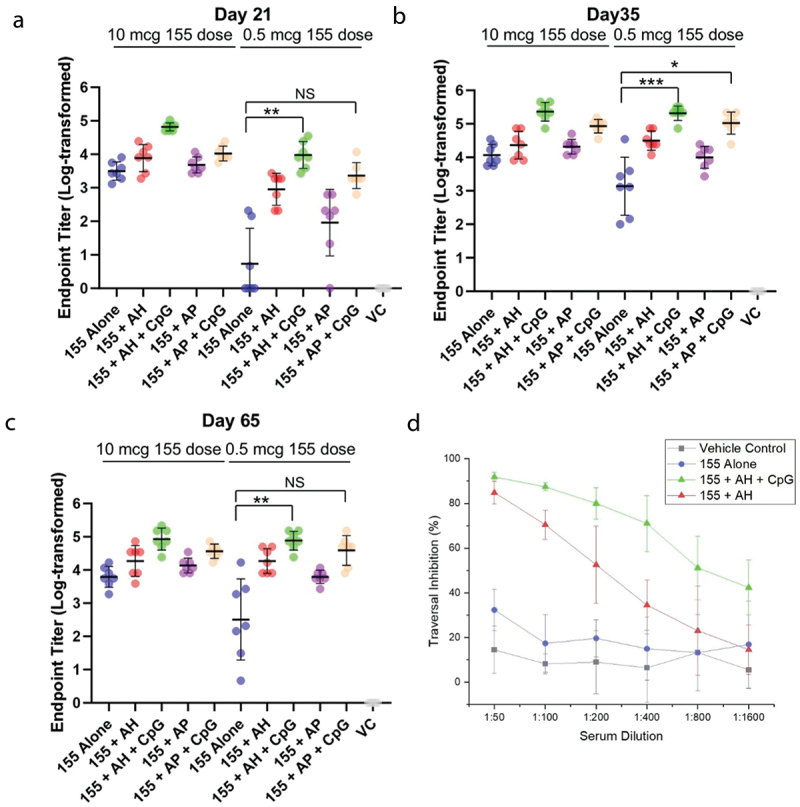
Mouse immunogenicity profiles for 155 antigen in the presence and absence of adjuvants (aluminum-salt and/or CpG) as measured by total antibody titers and serum sporozoite traversal inhibition. Mice were injected with either 0.2 or 0.01 mg/mL 155 antigen (10 or 0.5 mcg per dose) on day 0 (primed) and day 21 (boosted) and then endpoint titers were measured on (a) day 21, (b) day 35, and (c) day 65. The concentrations of the aluminum-salt adjuvants (AH, Alhydrogel™ or AP, Adju-Phos™) and CpG were 3.0 (150 mcg AH per dose) and 0.6 mg/mL (30 mcg CpG per dose), respectively. Error bars represent SD values from 7 mice per group. A vehicle control (VC) group of 6 mice were injected with HBS pH 7.0 buffer. Statistical significance (NS: not significant; *p* < .05 (*); *p* < .01 (**); *p* < .001(***)) was determined using a Kruskal-Wallis and Dunn’s multiple comparison test. (d) Sporozoite traversal percent inhibition values with serially diluted mouse serum from Day 35 of the vehicle control (HBS only, gray), 0.5 mcg 155 antigen alone (blue), or 0.5 mcg 155 antigen adsorbed to 3.0 mg/mL AH with (green) or without 0.6 mg/mL CpG (red). Error bars represent SD from each pooled serum analyzed in three independent experiments.

The immunogenicity profiles of unadjuvanted 155 antigen vs. various adjuvanted 155 antigen formulations were assessed in a mouse model. Two doses of the 155 antigen (10 or 0.5 mcg), either alone or formulated with one of the aluminum-salt adjuvants (AH, AP), with or without CpG, were evaluated. All samples were formulated immediately prior to administration (i.e., bedside mixed) and were injected using a prime (Day 0) and boost (Day 21) scheme. Mouse serum was collected 21, 35, and 65 days after priming and anti-PfCSP antibody endpoint titers (EPT) were measured. As shown in Figure 4a, most of the mice seroconverted by day 21 when immunized with the 155 antigen, while no immunoreactivity was measured in the vehicle control (HBS pH 7.0) group. In the mice that received 155 antigen, a wide range of EPTs were observed between the antigen doses and formulations. The largest difference between the two doses of the 155 antigen was observed on day 21 in which the EPTs of 0.5 mcg dose were 10^1^-10^3^ lower compared to similar formulations containing 10 mcg dose. By day 35 (Figure 4b) and 65 (Figure 4c), EPTs of the two 155 antigen doses were comparable for most formulations except for unadjuvanted 155 antigen, in which the EPT of 0.5 mcg dose was ~10^1^ lower at day 35 and 65 relative to 10 mcg dose of unadjuvanted antigen. Since the day 21 EPT with the higher dose appeared to already be close to maximizing the immune response, subsequent formulation comparisons are discussed using the results of the 0.5 mcg dose of the 155 antigen.

As expected, EPTs of unadjuvanted 155 antigen were the lowest of all 155 antigen formulations evaluated. The inclusion of either aluminum-salt adjuvant alone increased EPTs by ~10^1^ compared to unadjuvanted 155 antigen, although this trend was not statistically significant (*p* > .05). Although the EPTs of the 155 antigen adsorbed to AH (no CpG) trended higher compared to the 155 antigen in solution with AP (no CpG), they were not statistically significant (*p* > .05). The highest EPTs measured were with the 155 antigen formulated with one aluminum-salt and CpG adjuvants. Compared to the unadjuvanted 155 antigen, EPTs of 155+AP+CpG were significantly (*p* < .05) higher on day 35, and the 155+AH+CpG EPTs were significantly (*p* < .05–0.001) higher than unadjuvanted 155 antigen on days 21, 35, and 65. Although the EPTs for the 155 antigen adsorbed to AH with CpG trended higher on each day tested compared to 155 in solution with AP+CpG, they were not statistically significant (*p* > .05). Overall, these results indicated (1) the antibody response trended higher by approximately an order of magnitude in the presence of either aluminum-salt adjuvant compared to unadjuvanted 155 albeit the increases were not statistically significant (*p* > .05), (2) the immune response trended toward further amplification when CpG was co-formulated with either aluminum-salt adjuvant compared to 155 in the presence of either aluminum-salt alone; however, the increases were not statistically significant (*p* > .05), (3) immunogenicity of 155 antigen was overall independent of the relative percent in solution vs. adsorbed to an aluminum-salt adjuvant, and (4) 155+AH+CpG formulation consistently elicited a significantly (*p* < .05–0.001) higher immune response compared to unadjuvanted 155 antigen.

Next, the inhibitory activity of the mouse antibodies generated from the optimal 155 antigen formulation containing AH or AH+CpG adjuvants were compared to the unadjuvanted 155 antigen in a sporozoite traversal inhibition assay. To test the parasitic inhibition of 155-derived antibodies, serum from mice collected 35 d after immunization with the vehicle control (HBS alone), 0.5 mcg 155 antigen alone, or 0.5 mcg 155 antigen adsorbed to AH with or without CpG were compared (Figure 4d). Pf sporozoite traversal was largely inhibited at the lowest dilution factor (1:50) by the serum from mice immunized with 155+AH compared to the 155 antigen alone, which showed slightly elevated inhibition levels compared to the vehicle control. Serum from mice immunized with 155+AH+CpG inhibited Pf sporozoite traversal also at the 1:50 dilution; however, the serum from this two adjuvanted 155 antigen formulation was much more effective at inhibiting traversal as a function of serum dilution compared to 155+AH without CpG. These results indicate that the 155 antigen adsorbed to AH with CpG not only generated significantly high antibodies titers compared to unadjuvanted 155 antigen but elicited antibodies that could efficiently inhibit Pf sporozoite traversal.

### Stability profiles of 155 antigen formulated with or without adjuvants

A 3-month stability study was initiated with the lead candidate formulation of adjuvanted 155 antigen (155+AH+CpG) as well as with a back-up formulation (155+AP+CpG) to assess the stability profile of the 155 antigen at different temperatures, either bound to AH + CpG or in solution with AP and CpG. In addition to using competitive ELISA to measure the antigenicity of the 155 antigen in the two adjuvanted formulations, we also applied a previously developed “strong-forced” desorption assay to qualitatively assess the total amount of 155 antigen bound to AH.^22^ Strong-forced desorption utilizes extreme experimental conditions (e.g., incubation with a buffer containing a high phosphate concentration at pH 7.0 and SDS detergent for 10 min at 98°C) to denature the protein antigen, desorb from the surface of the aluminum-salt adjuvant, and then measure by SDS-PAGE analysis.

**Figure 5.**
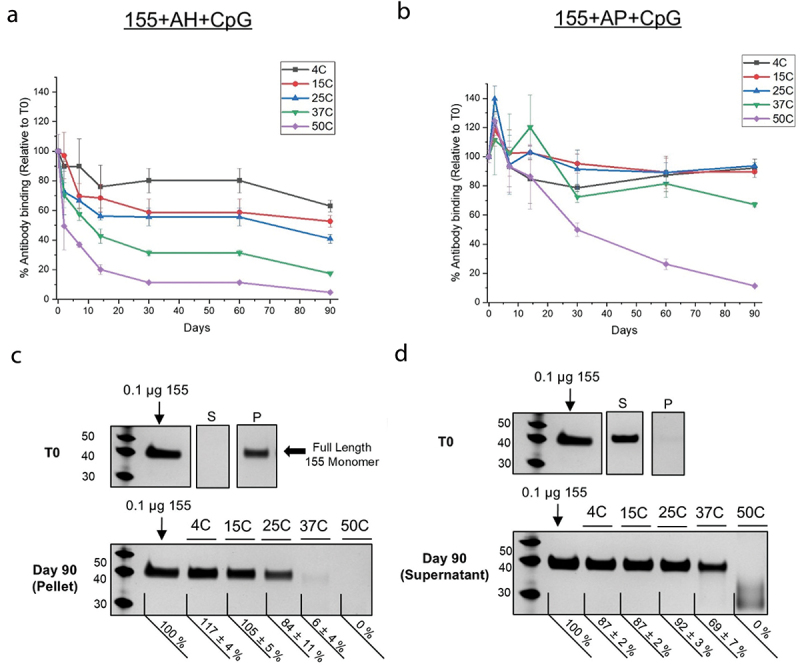
Storage stability profiles of formulated 155 antigen (with indicated adjuvants) at different temperatures as measured by competitive ELISA and SDS-PAGE. (A-B) Relative *in vitro* antibody binding values (percent vs time zero) as measured by competitive ELISA for the 155 antigen samples (a) adsorbed to AH with CpG, or (B) in solution with AP and CpG and stored at 4°, 15°, 25°, 37°, or 50°C up to 90 d. Error bars represent the range from *n* = 2 (two independent samples each measured once). (b) Representative SDS-PAGE gels (silver-stained, reduced) of (c) 155 antigen adsorbed to AH with CpG, or (d) 155 antigen in solution with AP and CpG at time 0 (T0) of the supernatant (S) or pellet (P) fractions following centrifugation, or after 90 d at 4–50°C (pellet fractions shown for 155+CpG+AH; supernatant fractions shown for 155+CpG+AP). Samples contained 0.01 mg/mL 155 antigen, 0.6 mg/mL CpG and 3.0 mg/mL alum (adsorbed to AH or in solution with AP) in HBS pH 7.0 buffer. Day 90 full length 155 antigen monomer band intensities from SDS-PAGE were quantified and listed relative to a 0.1 mcg 155 antigen control loaded on every gel. Error bars denote the range from *n* = 2 (two independent samples each measured once).

The antigenicity of 155 was distinct between two adjuvanted formulations (155+AH+CpG and 155+AP+CpG) stored under real-time (4°C), accelerated (15°, 25°, 37°C) and stressed (50°C) conditions over 3 months (Figures 5a,b). Relative to T0, anti-PfCSP antibody binding to the 155 antigen adsorbed to AH with CpG decreased primarily within the first 2–4 wks of storage and was more prominent as a function of increased storage temperature, which then decreased slightly over the next 8–10 wks. In contrast, small-to-no loss in antigenicity was observed for the 155 antigen in solution with AP and CpG when stored at 4, 15, 25, or 37°C after 3 months (less than ~25%). For the 155 antigen in the two formulations stored at 50°C, antigenicity steadily decreased over time and antibody binding was ~10% relative to T0 at ~1 month (155 adsorbed to AH + CpG) and ~3 months (155 antigen in solution with AP + CpG). Given the evidential multi-phasic decrease in anti-PfCSP antibody binding to the 155 antigen adsorbed to AH with CpG and the modest loss in antigenicity after 3 months for the 155 antigen in solution with AP and CpG at 4, 15, 25, or 37°C, additional stability studies (i.e., more timepoints, more replicates per timepoint, and longer incubation times) are recommended as part of future work to statistically quantify the real-time, accelerated, and stressed antigenicity slopes of this process to elucidate the storage stability of the 155 antigen in different formulations.

SDS-PAGE analysis was used to compare the integrity and adsorptive state of the 155 antigen (i.e., adsorbed to AH or in solution with AP) at T0 and after 90 d storage at each temperature using the “strong desorption” treatment described above. As expected at T0, the 155 antigen was detected essentially only in the pellet fraction (i.e., adsorbed) or supernatant fraction (i.e., in solution) in the presence of AH + CpG and AP + CpG formulations, respectively (Figures 5c,d). After storage for 3 months at 4°C, no change in the amount of the 155 antigen was observed with either formulation (i.e., adsorbed to AH+CpG or in solution with AP+CpG). Under accelerated storage conditions (15°, 25°, 37°C), however, changes were observed. With the 155+AH+CpG formulation, the intensity of the 155 antigen monomer band (as measured by percentage vs. a 0.1 mcg 155 antigen loading control) decreased as the temperature increased from 15° (105 ± 5%), 25° (84 ± 11%), 37° (6 ± 4%), and 50°C (none detected). In contrast, for the 155+AP+CpG formulation, after storage for 3 months at 15° or 25°C, the intensity of the 155 antigen monomer band was overall similar to T0. At higher temperatures, however, the monomer band was less intense at 37°C (69 ± 7%), while at 50°C, the monomer band was not observed and only lower MW smeared bands were present.

In summary, the following two conclusions can be made from these results. First, when the 155 antigen was stored bound to AH (+ CpG) adjuvants, the strength of antigen-adjuvant interactions increased over time as observed by both increasing inability to desorb the 155 antigen from AH and decreasing antibody binding readouts (see next section), especially as the storage temperature increased. Second, when the 155 antigen was stored in solution in the presence of AP and CpG adjuvants, the 155 antigen showed smaller losses of antibody binding (see next section) at lower temperatures, but at stressed temperatures (e.g., 50°C), antigen instability was observed due to loss of protein integrity. As a next step, we then investigated in more detail the nature of the interaction of the 155 antigen with AH adjuvant and then evaluated how both antibody binding and mouse immunogenicity readouts are affected during storage for both the AH+CpG and AP+CpG formulations of the 155 antigen.

### Better understanding of the interaction between 155 antigen and AH over time and its effect on mouse immunogenicity

To better understand the nature and strength of the binding interactions between the 155 antigen and AH adjuvant, we performed a previously developed “mild-forced” desorption assay.^22^ In this experiment, phosphate buffer anions are added to competitively displace AH-adsorbed proteins from the surface of the alum under relatively mild experimental conditions (e.g., 1 h at room temperature), which are expected to retain the protein antigen’s structural integrity.^22^ Since desorption is related to adsorptive strength, changes to the amount of protein desorbed from AH indicate a strengthening (less desorbed) or weakening (more desorbed) of the protein-AH interaction over time.^23,24^ To measure changes in the interaction between the 155 antigen and AH over time, 100 mm sodium phosphate pH 7.0 was added to 155+AH samples after storage at various temperatures/times, and the amount of desorbed the 155 antigen was determined by SDS-PAGE analysis. CpG was not included in this study due to material limitations, and a 10-fold higher concentration of the 155 antigen (0.2 mg/mL) was used compared to the stability study results described above to permit a more quantitative assessment of antigen-adjuvant using Coomassie staining (instead of the silver stain used previously at the lower protein dose) during these “mild-forced desorption” analyses.

**Figure 6.**
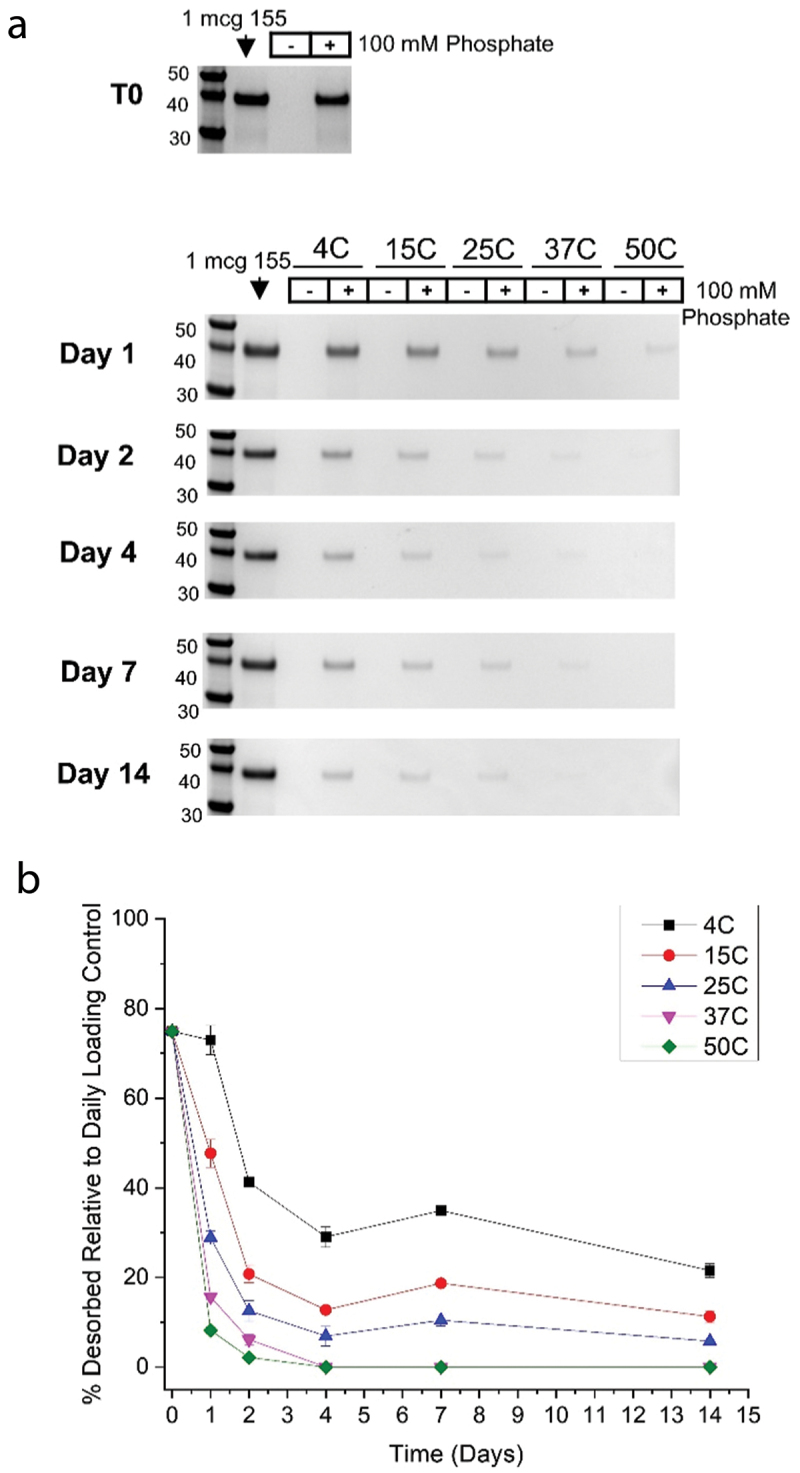
Increased binding interaction between 155 antigen adsorbed to AH adjuvant during storage as measured by ability to desorb the antigen using phosphate buffer. (a) Representative SDS-PAGE gels (Coomassie-stained, reduced) of the supernatant fraction of centrifuged samples of 0.2 mg/mL 155 antigen adsorbed to 3.0 mg/mL AH adjuvant in HBS pH 7.0 buffer at time 0 (T0), and over 14 d of storage at 4°, 15°, 25°, 37°, or 50°C. Samples were incubated with (+) or without (-) 100 mm phosphate pH 7.0 for 1 hr. At room temperature prior to centrifugation. (b) 155 antigen band intensities from SDS-PAGE were quantified and plotted relative to a 1 mcg 155 antigen control loaded on every gel. Error bars denote the range from *n* = 2 (two independent samples each measured once).

The gels displayed in Figure 6a show the intensity of the 155 antigen monomer band was less intense over time compared to time 0 (T0). The decreasing band intensity demonstrates it became more difficult to desorb the 155 antigen from AH during storage, and this effect was more pronounced with increasing storage temperature. As shown in Figure 6b, the relative percent of the 155 antigen desorbed from AH at T0 was ~80%, which decreased substantially within the first 2–4 d of storage at all temperatures, and then leveled off for the remainder of the 2 wks study. Given the brief duration of this study (2 wks with five timepoints), and the precision of SDS-PAGE band quantitation, additional stability studies (i.e., more timepoints, more replicates per timepoint, and longer incubation times out to months) are recommended as part of future work to statistically quantify the stability slopes of this process in different formulations. In summary, these initial results demonstrate the trend that the strength of the interaction(s) between the 155 antigen bound to AH adjuvant increases during storage as a function of both time and temperature (see Discussion).

**Figure 7.**
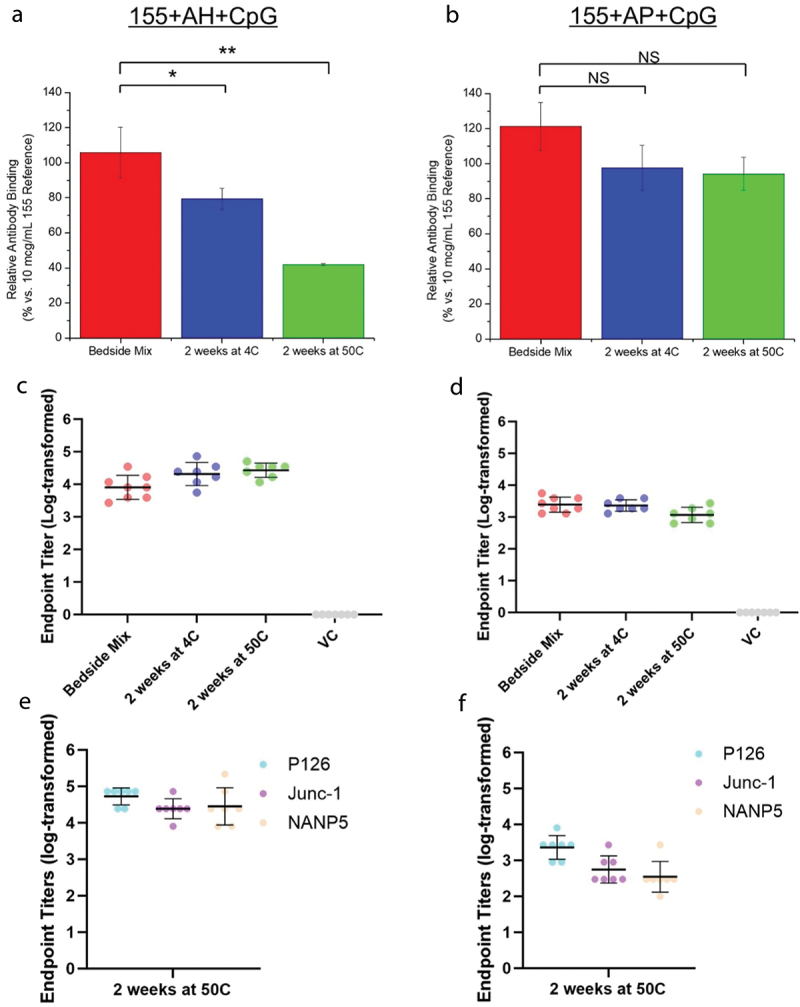
*In vitro* antigenicity vs. *in vivo* mouse immunogenicity profiles of formulated 155 antigen (with indicated adjuvants) before and after thermal stress exposure. Mean values of *in vitro* relative antibody binding to a 10 mcg/mL 155 antigen reference as measured by competitive ELISA for the 155 antigen (a) adsorbed to AH with CpG, or (b) in solution with AP and CpG. Samples were tested either immediately when formulated (bedside mix) or after 2 wks storage at 4° or 50°C. Error bars represent SD of *n* = 3–4 (three or four independent samples each analyzed in duplicate). Statistical significance (NS: not significant; *p* < .05 (*); *p* < .01 (**)) was determined using a Student’s test. (c-f) *In vivo* mouse immunogenicity results of the same formulated 155 antigen samples from serum 21 d after priming (*n* = 7 mice per group). (c, d) End-point titers of serum from indicated 155 antigen samples as measured against full-length PfCSP. A vehicle control (VC) injected with HBS pH 7.0 buffer is also shown. (e–f) End-point titers of serum from the 2 wk., 50°C samples tested using one of three CSP peptides (P126, Junc-1, NANP5). Samples contained 0.01 mg/mL 155 antigen (0.5 mcg per dose), 0.6 mg/mL CpG (30 mcg per dose) and 3.0 mg/mL alum (AH or AP as indicated, 150 mcg per dose) in HBS pH 7.0 buffer.

To better understand the effects of antigen-adjuvant interactions on 155 antigen integrity, the *in vitro* antigenicity and *in vivo* immunogenicity were evaluated for the two lead adjuvanted formulation (i.e., 155 antigen either adsorbed to AH with CpG or in solution with AP and CpG) during a short-term stability study (4° and 50°C for 2 wks). First, the ability of formulated 155 antigen (bound to AH + CpG) to bind the PfCSP specific-mAb, compared to freshly formulated (i.e., bedside mix) material, decreased significantly by ~25% (*p* < .05) or ~60% (*p* < .01) after 2 wks at 4° or 50°C, respectively (Figure 7a). Conversely, antibody binding to the formulated 155 antigen (in solution with AP and CpG) was not affected with no statistically significant differences (*p* > .05) observed under the same conditions compared to the bedside mix sample (Figure 7b).

Next, the *in vivo* immunogenicity of the same bedside mix and stability samples was evaluated in mice using the prime/boost/bleed scheme described above. Unlike the *in vitro* antigenicity results, no significant (*p* > .05) decrease in the *in vivo* EPTs was observed, as measured at Day 21 post injection, for the 155 antigen adsorbed to AH with CpG (Figure 7c) or in solution with AP and CpG (Figure 7d) stored for 2 wks at 4° or 50°C compared to the bedside mix sample. The EPTs of these adjuvanted 155 antigen samples all increased to 10^5^-10^6^ by day 35, following a boost injection on day 21, and remained at those EPTs until at least day 65, and the EPTs values were not significantly (*p* > .05) different from each other (data not shown).

We also investigated whether the antibody immune responses in mice elicited by the thermally stressed 155 antigen stability samples were more reactive toward the junction or NANP repeat epitopes comprising the PfCSP portion of 155 antigen. Three PfCSP peptides were tested with the mouse serum, which consisted of the PfCSP junction (Junc-1) or NANP repeat (NANP5) primary sequences, and a peptide (P126) that encompasses both Junc-1 and NANP5 epitopes.^17^ As shown in Figure 7e, for the 155 antigen bound to AH+CpG formulation after storage for 2 wks at 50°C, the reactivity of the mouse serum toward either PfCSP epitope alone (Junc-1 or NANP5) was comparable, and both trended slightly lower relative to the PfCSP peptide (P126), but the trends were not statistically significant (*p* > .05). As shown in Figure 7f, for the 155 antigen in solution formulated with AP+CpG, after storage for 2 wks at 50°C, the reactivity of the mouse serum toward either PfCSP epitope alone (Junc-1 or NANP5) was comparable, and both trended slightly lower, but were not statistically significant (*p* > .05), relative to the PfCSP peptide (P126). Although the reactivity of the 155+AH+CpG mouse serum was consistently higher toward the three PfCSP peptides compared to 155+AP+CpG, these trends were not statistically significant (*p* > .05). In summary, these results show that strengthening interaction between the 155 antigen bound to the AH adjuvant during storage causes an apparent loss in antigenicity (ability to bind an antigen-specific mAb while bound to the alum surface), yet there is no effect on *in vivo* performance in a mouse immunogenicity model (see Discussion).

## Discussion

### Adjuvanted formulations of current Malaria vaccines and the new 155 antigen vaccine candidate

Either one or multiple adjuvants are routinely combined with recombinant protein vaccine antigens to enhance and broaden immune responses.^25^ The two currently available, WHO-recommended malaria vaccines are both formulated with adjuvants: (1) RTS,S vaccine is formulated with the AS01 adjuvant consisting of liposomes, the TLR-4 agonist monophosphoryl lipid A, and the saponin QS21, and (2) R21 vaccine utilizes Matrix-M, a lipid nanoparticle adjuvant system derived from two saponin fractions that includes QS21.^2,10,26^ Both AS01 and Matrix-M adjuvant systems induce potent humoral and cellular responses,^10,27^ however, they are complex in composition, costly to produce, and limited in terms of manufacturing and commercial availability.^11,28^ Based on these considerations, this work focused on the optimization of low-cost aluminum-salt formulations of a new malaria vaccine candidate, either alone or in the presence of CpG oligonucleotides as a second adjuvant.

The 145S antigen, a predecessor to 155 antigen described in this work, was shown previously to induce a strong humoral response when formulated with the LMQ adjuvant system, which includes QS21 and a liposome-incorporated TLR-4 agonist 3D6AP.^17^ In the current study, we focused on the possibility of using one of the two aluminum-salt adjuvants (AH or AP) with the 155 antigen due to their well-established safety profile in adults, children and infants, as well as their low cost, wide availability, and ability to generate a primarily humoral immune response.^25,29^ A second adjuvant, CpG 1018®, was included in this study, as this TLR-9 agonist is commercially available and has been shown to elicit primarily cellular immune responses. Therefore, the addition of the CpG 1018® adjuvant could complement aluminum-salt adjuvants in inducing broader immunogenic responses when administered with the 155 antigen. The synergistic effect of combining aluminum-salt and CpG 1018® adjuvants has been demonstrated through multiple clinical and commercial COVID-19 vaccines.^20,30,31^

In this work, the highest antibody EPTs in mice were associated with 155 formulations containing one of the aluminum-salt adjuvants (AH or AP) and CpG. The immunogenicity of the 155 antigen alone (no adjuvants) was consistently lower than any formulation containing one or two adjuvants. The presence of either aluminum-salt adjuvant increased EPTs by 10–100-fold compared to the 155 antigen alone, although this trend was not statistically significant. In contrast, EPTs were significantly higher (*p* < .05–0.001) when the 155 antigen was formulated with either AH+CpG or AP+CpG adjuvant combinations compared to the 155 antigen alone. While these results demonstrate the synergistic effect of these two adjuvants, more in-depth studies on the nature of the immunological responses elicited (e.g., humoral vs cellular) by these formulations are required to better understand the contribution of each adjuvant. For example, a higher cellular (Th1) response compared a humoral (Th2) response would indicate a greater contribution of CpG toward the immune response compared to AH or AP, while a lower IgG2 response compared to IgG1 would suggest a weaker CpG contribution.^20,32^ The concentration of each of these adjuvants and their resulting contribution to the immune response toward the 155 antigen should also be evaluated in future studies, which could further support the inclusion of both adjuvants (aluminum-salt and CpG) given the inherent added costs and complexity of developing a co-adjuvanted vaccine candidate formulation. While the composition of the immune response was not determined in this study, antibodies generated from 155+AH or 155+AH+CpG both effectively inhibited Pf sporozoite traversal at the lowest serum dilution factor but 155+AH+CpG was much more effective at inhibiting traversal as a function of serum dilution compared to 155+AH without CpG. Pf sporozoite traversal was minimally inhibited in serum from mice that were administered 155 antigen alone.

In addition, we evaluated key pharmaceutical properties, including antigen-adjuvant interactions and stability profiles, of the different adjuvanted formulations of 155 antigen (*H. pylori* ferritin-based nanoparticle with multimeric display of the PfsCSP antigen; see Figure 1e). The 155 antigen was shown to completely adsorb to AH but remained in solution in the presence of AP adjuvants. The overall conformational stability of the 155 antigen is inherently high (i.e., Tonset value of 155 in solution is ~75°C) but was shown to be destabilized by the AH adsorption (~5°C lower Tonset value when bound to AH) but not destabilized by the AP addition (~3°C higher Tonset value in solution in the presence of AP). The presence of CpG had no measurable influence on the overall conformational stability of the 155 antigen or the ability to adsorb to AH. Next, during storage stability studies, different stability profiles of formulated 155 were revealed when comparing (1) adsorbed to AH and CpG vs. (2) in solution with AP and CpG.

For the 155+AH+CpG formulation (antigen bound to alum), the *in vitro* antigenicity and ability to desorb the 155 antigen from AH with CpG decreased as a function of storage temperature, particularly within the first 2 wks of storage. These *in vitro* changes, however, did not significantly (*p* > .05) alter the immune response to this malaria antigen in mice when comparing freshly formulated 155+AH+CpG vs. after 2 wks at 4° or 50°C. As described in the next section, the *in vitro* observations (decreased antigenicity and alum-desorption) are likely due to an increased binding interaction between the antigen and aluminum-salt adjuvant which do not affect *in vivo* performance.

For the 155+AP+CpG formulation (antigen in solution in the presence of the adjuvants), the *in vitro* antigenicity and the *in vivo* mouse immunogenicity of 155 after 2 wks at 4° or 50°C were not statistically different compared to a bedside mixed sample. After longer storage times at accelerated or stressed temperatures (i.e. 3 months at 37° or 50°C, respectively), however, the antigenicity of 155 decreased and a concomitant loss of protein integrity was observed. The mouse immunogenicity profiles of these 155+AP+CpG samples (3 months at 37° or 50°C) were not evaluated due to limited resources, and prioritization of the 155+AH+CpG formulation, however, would be of interest as part of future work.

### Analytical and formulation challenges encountered with 155 antigen vaccine candidate formulated with AH +CpG

While formulated 155 antigen with adjuvants (e.g., adsorbing the 155 antigen and CpG to AH) offers notable immunological benefits compared to unadjuvanted 155 antigen, this formulation composition imparts analytical challenges to ensure the quality and consistency of this adjuvanted 155 antigen-based malaria vaccine candidate. The inherent colloidal and chemical properties of aluminum-salt adjuvants limit the number of analytical techniques available for characterizing antigens adjuvanted with aluminum-salt adjuvant and has been thoroughly discussed elsewhere.^24,32^ In addition, the readouts from the limited analytical techniques available to characterize and/or monitor the structural integrity and physicochemical stability of aluminum-salt-adsorbed vaccine antigens (e.g., Fourier transform infrared spectroscopy, differential scanning calorimetry) would presumably not be informative with the 155 antigen since the generated signals would likely originate mostly from the apoferritin scaffold and less from the intrinsically disordered PfCSP region. As part of future work, the order/disorder of PfCSP region could potentially be elucidated through hydrogen-deuterium exchange mass spectrometry experiments,^33^ which we have recently demonstrated to successfully monitor the backbone flexibility of a different protein antigen adsorbed to AH.^34^

Another analytical challenge for a 155+AH+CpG formulation is the lack of concordance observed between *in vitro* antigenicity and *in vivo* immunogenicity with stressed 155 antigen adsorbed to AH with CpG (2 wks at 50°C), which potentially indicates that the structural alterations of the 155 antigen observed by the *in vitro* competitive ELISA assay (binding to a PfCSP-specific mAb) are not necessarily predictive of *in vivo* performance in mice. These observations (decreased *in vitro* antigenicity and unaffected *in vivo* immunogenicity) may indicate that the developed competitive ELISA assay is a sensitive probe of interactions between AH and the 155 antigen but may not be easily implemented as an *in vitro* method to measure potency of this malaria vaccine candidate.

Binding interactions between a protein antigen and AH can change over time, particularly within the first few days/weeks of formulation. Depending on the compounding strategy, the distribution of protein on the surface of aluminum-salt can vary substantially initially, but generally reach uniformity over time, a process known as “maturation.”^35^ In our studies, CpG was fully adsorbed to AH prior to adding the 155 antigen. Under these conditions, >75% of the 155 could be readily desorbed under “mild-forced” conditions and ~100% could be recovered by “strong-forced” desorption conditions. During real-time or accelerated storage conditions, however, less 155 antigen was desorbed using similar experimental conditions indicating an enhanced interaction between the malaria antigen and AH during storage.

The increased strength of interaction between the 155 antigen and AH adjuvant is likely due to the composition of the PfCSP region displayed on the surface of the 155 nanoparticle antigen. At the neutral pH of the formulation buffer used in these studies (HBS, pH 7.0), the positive surface charge of AH and the negative surface charge of 155 antigen, as determined by zeta potential measurements, suggest that the initial interaction occurs primarily via electrostatic charge–charge interactions, as opposed to the other potential protein-aluminum-salt adjuvant interactions known to occur (i.e., ligand exchange, hydrophobic interactions, etc.).^24^ The PfCSP region of the 155 antigen contains 7 charged amino acids (one Lys, one Glu, and five Asp) with a computationally predicted pI of 3.6. The high charge density of this (likely) intrinsically disordered and negatively charged region (24 protein monomers comprise a 155 nanoparticle antigen) multivalently displayed on the surface of the apo-ferritin scaffold could initially interact with AH through a small number of 155 antigen monomers displayed on the nanoparticle surface. Over time, an additional 155 antigen monomers displayed on the surface of the nanoparticle may be associated with more aluminum-salt particles, resulting in a stronger association between the antigen and aluminum-salt adjuvant and more resistant to our mild- and strong-forced desorption SDS-PAGE conditions.

The decreased *in vitro* antigenicity of the 155 antigen adsorbed to AH with CpG over time supports this hypothesis as the availability of epitopes recognized by the PfCSP mAb would be sterically hindered by the aluminum-adjuvant and result in decreased binding levels. To test this steric hinderance hypothesis, future work could be aimed at screening negatively charged excipients to pre-treat AH or co-formulate with the 155 antigen and AH to weaken the interaction between the antigen and aluminum-salt adjuvant. Future work could also be aimed at recovering the 155 antigen with reduced *in vitro* antigenicity from AH and utilizing liquid chromatography and mass spectrometry to assess if the PfCSP portion of the 155 antigen remained covalently attached to the apoferritin scaffold and was not chemically modified. The lack of a corresponding immunological decrease in mice compared to the *in vitro* antigenicity results suggests that the PfCSP epitopes in the 155 antigen remained intact and were accessible to elicit an immune response comparable with freshly formulated (i.e. bedside mix) material.

## Conclusions

In this work, different adjuvanted formulations of a malaria vaccine candidate termed 155 antigen were analytically characterized through a combination of *in vitro* physicochemical analyses, antigen-adjuvant binding studies, and storage stability studies as evaluated by both *in vitro* antigenicity (binding to a PfCSP-specific mAb) and *in vivo* immunogenicity profiles in mice. The recombinant glyco-engineered apoferritin nanoparticle with multimeric display of PfCSP antigens was shown to be pure and overall homogenous, with expected primary structure, post-translational modifications, and particle size along with no aggregation and high conformational stability. The 155 antigen adsorbed to one aluminum-salt adjuvant (Alhydrogel^TM^, AH) but remained in solution in the presence of a second aluminum-salt (Adju-Phos^TM^, AP). The CpG 1018® (CpG) adjuvant followed a similar adsorption pattern to these two aluminum-salt adjuvants as the 155 antigen.

For assessing potency of the 155 antigen formulations, *in vitro* antigenicity was determined by measuring the binding to a PfCSP-specific mAb using competitive ELISA, and *in vivo* immunogenicity was evaluated in mice. Mouse immunogenicity studies indicated 10 or 0.5 mcg of 155 antigen, prepared in different adjuvanted formulations, all elicited a potent total anti-PfCSP antibody response (compared to 155 antigen alone). The most notably enhanced adjuvanted 155 antigen formulation contained both AH + CpG adjuvants, and the immune sera elicited by this formulation was shown to inhibit hepatocyte traversal substantially more compared to that elicited by 155 antigen alone. Although stability studies with 155+AH+CpG formulation showed strengthened antigen-adjuvant interactions over time, especially at elevated temperatures, similar immunological responses in mice were observed in these stability samples compared to freshly adsorbed material (bedside mix), indicating the structural integrity of the key epitopes of the 155 antigen remained intact. In total, these results establish the recombinant 155 antigen as a homogeneous and well-characterized protein, and when formulated with commonly used and low-cost aluminum-salt and CpG 1018® adjuvants, from both a pharmaceutical and immunological perspective, is a promising malaria vaccine candidate that warrants further pre-clinical and eventually clinical testing.

## Supplementary Material

Hickey et al 2024 Supplemental Section Revision Final Clean.docx

## Data Availability

All data associated with this study are available with the corresponding author(s). The datasets generated and/or analyzed are also available in the KU ScholarWorks repository https://doi.org/10.17161/1808.35808.

## Abbreviations

AH: Alhydrogel^TM^
AP: Adju-Phos^TM^
CpG: CpG 1018**®**
CSP: Circumsporozite protein
DLS: Dynamic light scattering
DSC: Differential scanning calorimetry
DTT: Dithiothreitol
ELISA: Enzyme-linked immunosorbent assay
EPT: End-point titer
HBsAg: Hepatitis B surface antigen
HBS: HEPES-buffered saline
IAM: Iodoacetamide
Pf: *Plasmodium falciparum*
PfCSP: *Plasmodium falciparum* circumsporozite protein
PBS: Phosphate-buffered saline
SDS-PAGE: Sodium dodecyl sulfate polyacrylamide gel electrophoresis
T0: Time zero
TLR: Toll-like receptor
Tm: Thermal melting temperature
Tonset: Thermal onset temperature
WHO: World Health Organization
