# Supplementary material for: PfCSP-ferritin nanoparticle malaria vaccine antigen formulated with aluminum-salt and CpG 1018® adjuvants: Preformulation characterization, antigen-adjuvant interactions, and mouse immunogenicity studies
Source: Hum Vaccin Immunother. 2025 Feb 4;21(1):2460749. doi: 10.1080/21645515.2025.2460749 (PMC11796538; doi:10.1080/21645515.2025.2460749)
Supplement: Hickey et al 2024 Supplemental Section Revision Final Clean.docx [file KHVI_A_2460749_SM5160.docx]

**SUPPLEMENTAL SECTION**

**PfCSP-Ferritin Nanoparticle Malaria Vaccine Antigen Formulated with Aluminum-salt and CpG 1018^®^ Adjuvants: Preformulation Characterization, Antigen-Adjuvant Interactions, and Mouse Immunogenicity Studies**

John M. Hickey^1^, Nitya Sharma^1^, Max Fairlamb^1^, Jennifer Doering^2^, Yetunde Adewunmi^2^, Katherine Prieto^3^, Giulia Costa^4^, Benjamin Wizel^5^, Elena A. Levashina^4^, Nicholas J. Mantis^2^, Jean-Philippe Julien^3, 6^, Sangeeta B. Joshi^1^, and David B. Volkin^1,*^

^1^ Department of Pharmaceutical Chemistry, Vaccine Analytics and Formulation Center, University of Kansas, Lawrence, KS, USA

^2^ Division of Infectious Disease, Wadsworth Center, New York State Department of Health, Albany, NY, USA

^3^ Program in Molecular Medicine, The Hospital for Sick Children, Research Institute, Toronto, ON, Canada

^4^ Vector Biology Unit, Max Planck Institute for Infection Biology, Berlin, Germany

^5^ Dynavax Technologies Corporation, Emeryville CA, USA

^6^ Departments of Biochemistry and Immunology, University of Toronto, ON, Canada

*Correspondence to: David B. Volkin: Multidisciplinary Research Building, 2030 Becker Drive, Lawrence, KS 66047, Email: volkin@ku.edu; Phone: 785-864-6262; Fax: 785-864-5736

**Key words:** Malaria Vaccine, Alhydrogel, Adju-Phos, CpG 1018**^®^**, Adjuvant, PfCSP, Immunogenicity, Formulation, Stability

**Supplemental Methods**

*155 Antigen Expression and Purification*- The 155 antigen is similar in sequence to the previously described 145S antigen, which consists of the PfCSP junction epitope, NVDP and NANP motifs, a 15-aa linker, H. pylori apoferritin, a 3-aa linker and PADRE, in addition to the introduction of non-native N-linked glycan sites into the H. pylori apoferritin scaffold at positions 79 and 99 ^1^. This next generation construct however differs from the 145S antigen by three main differences: 1) the 145S antigen contains a N-terminal Strep-II tag followed by a TEV cleavage site; the 155 antigen does not. 2) the 145S antigen is purified via StrepTrap HP (Cytiva) chromatography followed by TEV cleavage of the Strep-II tag for 3 h at room temperature, followed by further purification via an additional StrepTrap HP (Cytiva) chromatography step and size exclusion chromatography; the 155 antigen is purified by more standard manufacturing techniques: anion exchange chromatography (HiTrap Q HP, Cytiva, Marlborough, MA), followed by hydrophobic interaction chromatography (HiTrap Phenyl HP, Cytiva), and 3) both antigens used in immunization studies contain high-mannose glycans: the 145S antigen by expression in HEK 293 GnT I-/- cells, and the 155 antigen by expression in a CHO cell line associated with more robust manufacturability with simultaneous addition of the α-Mannosidase I inhibitor kifunensine in the media during expression.

The 155 antigen ^1^was expressed in a stable pool of CHO-M cells with kifunensine by Selexis SA. After harvesting, the supernatant was filtered and buffer-exchanged into 20 mM Sodium Acetate pH 4 using a tangential flow filtration LV Centramate Cassette of 100 kDa molecular weight cut-off (Pall Corporation, New York, NY). Purification was performed by anion exchange chromatography (HiTrap Q HP), followed by hydrophobic interaction chromatography (HiTrap Phenyl HP, Cytiva) with 1.5 M ammonium sulfate. The purified 155 antigen fractions were desalted into 1X PBS buffer, sterile-filtered and frozen at -80°C in 1.5 mL Eppendorf tubes.

*SDS-PAGE*- Prior to SDS-PAGE analysis, samples containing 155 antigen alone or in the presence of various adjuvants (CpG, AH, AP) were centrifuged at 17,000 x g to separate in solution (supernatant) and alum-adsorbed (pellet) fractions. The supernatant fraction was transferred to a new Eppendorf tube, mixed with 50 mM Dithiothreitol (DTT, Thermo Fisher Scientific, Waltham, MA) and LDS buffer (Invitrogen, Waltham, MA) and then heated for 10 min at 98°C. For non-reducing conditions, 20 mM iodoacetamide (IAM, Thermo Fisher Scientific) was mixed with the supernatant fraction for 15 min at room temperature prior to mixing with LDS buffer and heating for 10 min at 98°C. 1 or 0.1 mcg 155 antigen was loaded in a NuPAGE^TM^ 4-12% Bis-Tris gel (Invitrogen) and then separated for 60 min at 125 V. A 1 or 0.1 mcg bulk 155 antigen reference sample was prepared daily and included with experimental 155 samples for quantification. The gel was then stained either using R250 Coomassie (Teknova, Hollister, CA) or silver-stain (Thermo Fisher Scientific) for 1 or 0.1 mcg 155 antigen, respectively. Gels were scanned using FluoroChem E (Protein Simple, Santa Clara, CA) gel imaging system and analyzed using AlphaView SA software (Protein Simple).

The pellet fraction was treated using mild- or strong-forced desorption conditions as described previously ^2^. For mild forced desorption, 0.1 M sodium phosphate pH 7.0 was mixed with the pellet for 1 hr. at room temperature, centrifuged for 5 min at 17,000 x g, and the supernatant was subjected to reducing SDS-PAGE conditions as described above. For strong forced desorption, the pellet was mixed with 0.4 M sodium phosphate, pH 7.0, 50 mM DTT, and LDS buffer, and then incubated for 10 min at 98°C. The sample was centrifuged for 5 min at 17,000 x g and the supernatant fraction was loaded into the SDS-PAGE gel.

*Intact Protein Mass Spectrometry*- Prior to intact mass analysis, bulk 155 antigen was diluted to 0.5 mg/mL using water and then centrifuged for 5 min at 17,000 x g. One mcl (0.5 mcg 155) was injected onto a Acquity Premier CSH-C18 column (2.1 x 150 mm, 1.7 mcm, Waters Corporation, Milford, MA) maintained at 50°C in a 1290 Infinity LC system (Agilent Technologies, Santa Clara, CA). 155 antigen was eluted from the column using a LC gradient of 20-40% B (A: LC-MS grade water with 0.1% formic acid, and B: LC-MS grade acetonitrile with 0.1% formic acid) over 20 min at a flow rate of 0.2 mL/min. The intact mass of the 155 antigen monomer was then measured using an in-line 6545XT QTOF system (Agilent Technologies) using a 290°C gas temperature, 4000V Vcap, and 180V fragmentor. Mass spectra were collected from 600-2200 m/z at 1 spectra/sec and processed using MassHunter (Agilent Technologies) with a deconvolution range of 10-50 kDa using a mass step of 1 Da.

*Differential Scanning Calorimetry (DSC)*- DSC analysis was performed using a MicroCal Auto-VP DSC-II equipped with a tantalum sample and reference cells (Malvern Panalytical, Malvern, UK). Samples were loaded into a DSC autosampler tray held at 5°C. Scans were taken from 20-120°C at a scan rate of 60°C/hour. The concentration of the 155 antigen was 0.4 mg/mL and for adjuvanted formulations, AH or AP was 3.0 mg/mL and CpG was 0.6 mg/mL. All samples were analyzed in duplicate. Origin software was used for background (HBS +/- AH or AP +/- CpG) subtraction and concentration normalization.

*Dynamic Light Scattering (DLS) and Zeta Potential*- DLS was performed using the DynaPro® Plate Reader ™ II (Wyatt Technology, Santa Barbara, CA). Bulk 155 antigen was diluted with water and 10X HBS to 0.2 mg/ml, and 30 mcL was added to a 384-well plate (Corning, Glendale, Arizona). DLS was performed at 25°C with an acquisition time of 5 sec and a total of 5 acquisitions. The hydrodynamic diameter of each sample was estimated after analyzing the intensity autocorrelation function via the multimodal size distribution method. For zeta potential analysis, bulk 155 antigen was diluted to 1.0 mg/mL using HBS pH 7.0 and zeta potential was measured using 10 runs (10 cycles per run) with a ZetaPALS analyzer (Brookhaven Instruments Corporation, NY). Zeta potential measurements were performed in duplicate.

*Competitive ELISA* **-**The antigen-antibody binding assay (binding of formulated 155 antigen to 4493 mAb) was adapted from procedure developed for other adjuvanted protein antigens as described in Jerajani *et. al.* 2022 ^3^. The assay was optimized for several parameters such as linearity, precision, accuracy, and stability indication for quantitation of the 155 antigen at 0.2 or 0.01 mg/mL, either unadjuvanted or in the presence of 3.0 mg/mL AH or AP +/- 0.6 mg/mL CpG. Using checkerboard titration, the final concentrations of coating antigen (bulk 155 antigen), experimental sample containing the 155 antigen, primary capture antibody (4493) and the secondary detection antibody dilutions were optimized to be a target concentration of 0.25 mcg/mL, 0.2 mcg/mL, 0.06 mcg/mL and 1:5000 dilution, respectively. The incubation times of the primary capture antibody binding, secondary detection antibody, tetramethylbenzidine (TMB) substrate and stop solution (1N HCl) were also optimized.

On the first day, ELISA plates (Corning) were coated overnight at 4°C with 155 antigen diluted to 0.25 mcg/ml in DPBS (Thermo Fisher Scientific). For each formulation of unadjuvanted or adjuvanted 155, a fresh reference standard with a similar formulation composition to an experimental sample that was prepared daily. On the same day, each reference standard and experimental sample were diluted with blocking buffer (1% Casein in TBS (Thermo Scientific) with 0.05% PS20 (Fisher Scientific)) such that the final concentration of 155 was 0.2 mcg/ml. The diluted reference standard and experimental samples were then incubated for 1-1.5 hrs. at room temperature with constant vertical rotation. The reference and experimental samples were serial diluted vertically in the PCR plate to create a range from 0.2 mcg/mL to 0.004 mcg/mL with a 1.75 dilution factor. Next, an equal volume of a PfCSP-specific mAb (4493 at 0.06 mcg/ml) was added to all the wells, the plates were then tightly capped with flat cap strips (Fisherbrand^TM^) and then rotated vertically at low speed overnight at room temperature.

The following day, the PCR plates containing the 155 antigen reference standard and experimental samples were centrifuged at 1,600 x g for 3 min. The 155 antigen coated ELISA plates were washed (1X PBS, 0.05% PS20) five times, and then blocked with blocking buffer for 1 hr. at room temperature. The ELISA plates were washed twice, and the supernatant from the centrifuged PCR plate was transferred to the ELISA plate. The ELISA plates were then incubated for 1.5-2 hrs. at room temperature with gentle shaking (300 rpm). The plates were washed five times, and goat-anti-human IgG-HRP secondary antibody (Invitrogen) diluted 1:5000 in the blocking buffer was added and the plate was incubated for 1 hr. at room temperature. The ELISA plates were then washed five times, TMB (Millipore Sigma, Burlington, MA) was added, and the plates were incubated in the dark. After 9 min, 1N HCl was added, and the plates were read at 450 nm using a SpectraMax M5 microplate reader (Molecular Devices, San Jose, CA). The binding data were analyzed using a four-parameter logistic (4 PL) equation (using a no weighting fit) as described previously ^3^.

*Preparation of Unadjuvanted and Adjuvanted 155 Antigen Samples*- For unadjuvanted samples, bulk 155 antigen was diluted with water and 10X concentrated formulation buffer to achieve a final concentration of 0.2 or 0.01 mg/ml 155 in HBS buffer (10 mM HEPES, 150 mM NaCl, pH 7.0). For adjuvanted samples, 0.6 mg/mL CpG was mixed with 10X concentrated HBS, 3.0 mg/mL AH or AP. Following incubation for 1 hr. at room temperature, 155 antigen was added to a final concentration of 0.2 or 0.01 mg/ml, and the solution was incubated for another hr. at room temperature. For both the 2 wk. *in vitro* antigenicity vs. *in vivo* immunogenicity study and the 3 month stability study, 0.01 mg/mL 155 antigen adjuvanted samples were prepared in 0.5 mL LoBind Eppendorf tubes (100 mcL sample per tube) and the lids were tightly covered with parafilm. Samples for the 2 wk. study (made in duplicate) were analyzed by competitive ELISA either immediately after formulating (bedside mix) or after 2 wks. of storage at 4 or 50°C. Samples for the 3 month stability study (made in duplicate) were then incubated at 4, 15, 25, 37, or 50°C and analyzed by competitive ELISA on T0, day 2, day 7, day 15, day 21, day 60 and day 90. Strong-forced desorption SDS-PAGE with silver-stain was performed with adjuvanted 155 antigen samples on T0 and Day 90. For the 2 wk. AH-adsorption study, 0.2 mg/mL 155 antigen with 0.6 mg/mL CpG and 3.0 mg/mL AH was incubated at 4, 15, 25, 37, or 50°C in 1.5 mL LoBind Eppendorf tubes (200 mcL sample per tube) and analyzed by mild-forced desorption SDS-PAGE (described above) on time 0 (T0), day 1, day 2, day 4, day 7, and day 14.

*Mouse Immunogenicity Studies*- Prior to mouse immunogenicity studies, 0.2 (10 mcg dose) or 0.01 mg/mL (0.5 mcg dose) 155 antigen was formulated with or without adjuvants (3.0 mg/mL AH or AP, 0.6 mg/mL CpG) as described above either immediately prior to administration (i.e., bedside mix) or after incubation at 4 or 50°C for 2 wks. The 155 antigen doses tested were based on mouse immunogenicity studies with a previous iteration of the antigen (145S) ^1^, while the doses of each adjuvant (AH, AP, CpG) were selected based on previous reports by others ^4, 5^ as well as our labs ^6-8^ with various recombinant protein antigens. Mouse studies were conducted in compliance with Wadsworth Center’s Institutional Animal Care and Use Committee (IACUC). Female C57BL/6 mice aged 6-8 wks. were obtained from The Jackson Laboratory (Bar Harbor, ME) and housed under conventional, specific-pathogen-free conditions. Mice were vaccinated subcutaneously on day 0 (prime) and day 21 (boost) with 50 mcl of formulations containing unadjuvanted or adjuvanted 155 antigen (n = 7-8) or HBS alone (vehicle control, VC, n = ^6^). Blood was collected from mice via the submandibular vein on days 21, 35, and 65.

*End Point Titer Analysis-* Direct ELISAs were performed by coating 384-well plates (Thermo Fisher Scientific) with full length recombinant PfCSP or peptides of P126, NANP5, or N-Junc1 (2 mcg/ml in PBS, see Results section for their description) and incubating overnight at 4°C. Plates were washed with PBS with 0.05% (v/v) Tween-20 (PBS-T), and then blocked for 2 hrs. with PBS-T containing 4% (v/v) bovine serum albumin (Millipore Sigma). Three-fold serial dilutions of serum (starting at 1:100) were then applied to the plate for 90 min at room temperature, washed and detected with HRP-conjugated goat anti-mouse IgG (Jackson ImmunoResearch, West Grove, PA). The ELISA plates were reveled using ABTS solution (Millipore Sigma) and analyzed using a SpectraMax iD3 spectrophotometer equipped with Softmax Pro 7.1.0 software (Molecular Devices). The endpoint titer was defined as the minimal dilution whose absorbance (405 nm) was > 3 times background, with background being defined as the average absorbance produced by wells with buffer alone.

*Pf Sporozoite Hepatocyte Traversal Assay-* Mouse sera were collected 35 days after immunization and pooled equivolumetrically for each treatment group (n=7): vehicle control (HBS only, no 155), 0.5 mcg 155 in HBS alone, or adsorbed to 3.0 mg/mL AH with 0.6 mg/mL CpG. The sporozoite traversal inhibition assay was performed as previously described in three independent biological experiments ^1^. Briefly, A. coluzzii mosquitoes ^9^ were infected with mature NF54 Pf gametocytes via artificial midi-feeders (Glass Instruments, the Netherlands) for 15 min and kept at 26°C and 80% humidity in a controlled S3 facility in accordance with local safety authorities (Landesamt für Gesundheit und Soziales Berlin, Germany, LAGeSo, project number 297/13). Infected mosquitoes received an additional uninfected blood meal 8 days post-infection and were collected for sporozoite isolation at 14-15 days post-infection. The human hepatocyte cell line HC-04 ^10^ was cultured at 37°C and 5% CO2 in HC-04 medium as previously described ^1^. One day before the assay, 60,000 cells were seeded in flat-bottomed 96-well plates (Corning). Sporozoites were isolated by grinding mosquito thoraces containing the salivary glands with glass pestles. The extracts were filtered with a 40 μm cell strainer (BD Biosciences). The isolated salivary gland Pf sporozoites were enumerated in a Malassez hemocytometer and 50,000 sporozoites in HC-04 medium were pre-incubated with serially diluted pooled serum samples for 30 min on ice as detailed in Figure 4D. Dextran-Tetramethylrhodamine (0.5 mg/mL, 10,000 MW, Molecular Probes) was added to the sporozoite suspensions, and the samples were transferred to the HC-04 cells in 50 µl final volume and incubated for 2 h at 37 °C and 5% CO2. HC-04 cells were washed, trypsinized and fixed with 1% PFA in PBS before flow cytometry quantification using a FACS LSR II instrument (BD Biosciences). Data analysis was performed using FlowJo 10.10.0 and for each sample traversal efficiency was calculated by subtraction of the background (dextran positivity in cells with uninfected mosquito salivary gland material). Traversal inhibition (%) was calculated as: 100 - (traversal efficiency SAMPLE / traversal efficiency COMPLETE MEDIUM x 100).

*Statistical Analyses-* A Student’s t-test was used to statistically compare in vitro antigenicity (competitive ELISA) of 155 antigen formulations. Endpoint titers of mouse sera were statistically analyzed using a Kruskal-Wallis test and Dunn’s multiple comparison test. For all statistical tests, the level of significance was defined as either not significant (NS) or statistically significant using three levels (p < 0.05 (*); p < 0.01 (**); p < 0.001(***)).

**Supplemental References**

1. Ludwig J, Scally SW, Costa G, et al. Glycosylated nanoparticle-based PfCSP vaccine confers long-lasting antibody responses and sterile protection in mouse malaria model. NPJ Vaccines 2023;8(1):86. <https://doi.org/10.1038/s41541-023-00687-x>.

2. McAdams D, Estrada M, Holland D, et al. Concordance of in vitro and in vivo measures of non-replicating rotavirus vaccine potency. Vaccine 2022;40(34):5069-5078. <https://doi.org/10.1016/j.vaccine.2022.07.017>.

3. Jerajani K, Wan Y, Hickey JM, et al. Analytical and Preformulation Characterization Studies of Human Papillomavirus Virus-Like Particles to Enable Quadrivalent Multi-Dose Vaccine Formulation Development. J Pharm Sci 2022;111(11):2983-2997. <https://doi.org/10.1016/j.xphs.2022.07.019>.

4. Vecchi S, Bufali S, Skibinski DAG, O'hagan DT, Singh M. Aluminum adjuvant dose guidelines in vaccine formulation for preclinical evaluations. J Pharm Sci-Us 2012;101(1):17-20. <https://doi.org/10.1002/jps.22759>.

5. Pollet J, Strych U, Chen WH, et al. Receptor-binding domain recombinant protein on alum-CpG induces broad protection against SARS-CoV-2 variants of concern. Vaccine 2022;40(26):3655-3663. <https://doi.org/10.1016/j.vaccine.2022.05.007>.

6. Bajoria S, Kaur K, Kumru OS, et al. Antigen-adjuvant interactions, stability, and immunogenicity profiles of a SARS-CoV-2 receptor-binding domain (RBD) antigen formulated with aluminum salt and CpG adjuvants. Hum Vaccin Immunother 2022;18(5):2079346. <https://doi.org/10.1080/21645515.2022.2079346>.

7. Bajoria S, Kumru OS, Doering J, et al. Nanoalum Formulations Containing Aluminum Hydroxide and CpG 1018(TM) Adjuvants: The Effect on Stability and Immunogenicity of a Recombinant SARS-CoV-2 RBD Antigen. Vaccines (Basel) 2023;11(6). <https://doi.org/10.3390/vaccines11061030>.

8. Kumru OS, Bajoria S, Kaur K, et al. Effects of aluminum-salt, CpG and emulsion adjuvants on the stability and immunogenicity of a virus-like particle displaying the SARS-CoV-2 receptor-binding domain (RBD). Hum Vaccin Immunother 2023;19(2):2264594. <https://doi.org/10.1080/21645515.2023.2264594>.

9. Harris C, Lambrechts L, Rousset F, et al. Polymorphisms in Anopheles gambiae immune genes associated with natural resistance to Plasmodium falciparum. PLoS Pathog 2010;6(9):e1001112. <https://doi.org/10.1371/journal.ppat.1001112>.

10. Sattabongkot J, Yimamnuaychoke N, Leelaudomlipi S, et al. Establishment of a human hepatocyte line that supports in vitro development of the exo-erythrocytic stages of the malaria parasites Plasmodium falciparum and P. vivax. Am J Trop Med Hyg 2006;74(5):708-715.
